# The Chemical Composition of *Scaptotrigona mexicana* Honey and Propolis Collected in Two Locations: Similarities and Differences

**DOI:** 10.3390/foods12173317

**Published:** 2023-09-03

**Authors:** Dessislava Gerginova, Milena Popova, Ralitsa Chimshirova, Boryana Trusheva, Maggie Shanahan, Miguel Guzmán, Erik Solorzano-Gordillo, Estefhanía López-Roblero, Marla Spivak, Svetlana Simova, Vassya Bankova

**Affiliations:** 1 Bulgarian NMR Centre, Institute of Organic Chemistry with Centre of Phytochemistry, Bulgarian Academy of Sciences, Acad. G. Bonchev Str., Bl. 9, 1113 Sofia, Bulgaria; dessislava.gerginova@orgchm.bas.bg; 2 Laboratory Chemistry of Natural Products, Institute of Organic Chemistry with Centre of Phytochemistry, Bulgarian Academy of Sciences, Acad. G. Bonchev Str., Bl. 9, 1113 Sofia, Bulgaria; milena.popova@orgchm.bas.bg (M.P.); ralitsa.chimshirova@orgchm.bas.bg (R.C.); boryana.trusheva@orgchm.bas.bg (B.T.); 3 Department of Entomology, University of Minnesota, 219 Hodson Hall, 1980 Folwell Ave., St. Paul, MN 55108, USA; mshanaha@umn.edu (M.S.); spiva001@umn.edu (M.S.); 4 El Colegio de la Frontera Sur, Carretera Antiguo Aeropuerto km 2.5, Tapachula 30700, Chiapas, Mexico; mguzman@ecosur.mx (M.G.); ejsolorzano@ecosur.edu.mx (E.S.-G.); el.roblero@outlook.es (E.L.-R.)

**Keywords:** stingless bees, *Scaptotrigona mexicana*, honey, propolis

## Abstract

The chemical composition of stingless bee honey and propolis depends on the plant sources they are derived from, and thus reflects the flora available in the vicinity of the hives, the preferences of the bee species, and the climate (altitude and temperature). To understand the relative influence of these factors, we studied the composition of honey and propolis of the stingless bee *Scaptotrigona mexicana*. Samples from 24 colonies were analyzed: 12 each from two *S. mexicana* meliponaries located in the state of Chiapas in southern Mexico, approximately 8.5 km apart, Tuxtla Chico and Cacahoatán. The chemical composition of honey and propolis was studied using nuclear magnetic resonance (NMR) and gas chromatography-mass spectrometry (GC-MS), respectively. The antioxidant activity of propolis was also studied. Chemometric analyses were applied. The Tuxtla Chico honey samples contained higher concentrations of glucose and fructose, while the Cacahoatán samples displayed a rich composition of di- and trisaccharides. These differences can be attributed to the distinct nectar sources utilized by the bees at each location. Propolis compositions in the two locations also demonstrated qualitative differences, indicating a specific choice of resins by the bees. The observed substantial variations in the chemical composition of propolis and honey of *S. mexicana* from two locations relatively close to each other supports the assumption that bee species cannot be considered the most important factor in determining their chemistry.

## 1. Introduction

Stingless bees, of the Meliponini tribe within the Apidae family, share a close genetic relationship with the more familiar Western honeybees, *Apis mellifera*. They earn their moniker “stingless” due to their greatly diminished stingers, which they do not employ for defense. Instead, they safeguard their nests through biting [1]. The Meliponini tribe encompasses a vast array of over 600 species distributed across tropical regions worldwide. Their highest numbers and diversity are found in the Neotropics (comprising South and Central Americas), as well as in tropical Africa, Southeast Asia, and Australia [2]. These bees display eusocial behavior and establish colonies led by a single queen, with worker populations ranging from a few dozen to several thousand [3].

The role of stingless bees in plant pollination carries substantial significance, as they contribute to the pollination of an estimated 40–90% of native and cultivated species in tropical regions [3]. The practice of domesticating stingless bees and utilizing their products dates back to pre-Colombian times in the Americas. The Mayan culture, for instance, engaged in meliponiculture, not only for sustenance and medicinal purposes, but also for the honey and propolis produced, which played a role in religious ceremonies. In regions such as Southeast Asia, Africa, and Australia, stingless beekeeping is a more recent development but is steadily gaining popularity. The products derived from stingless bees are emerging as a viable market in various parts of the world, with the potential for meliponiculture to serve as an economic and social support in forested areas and regions facing significant challenges [4].

In Mexico, 46 species of stingless bees occur; many of these are cultivated for the production of honey, pollen, and propolis [5]. Stingless beekeeping, or meliponiculture, is part of a rich biocultural tradition since ancient times [6]. *Scapotrigona mexicana* Guérin-Méneville was preserved by pre-Columbian societies in Mexico and is still kept in many regions today [7]. These bees are important pollinators of many wildflowers and crops and are of both ecological and economic importance [8].

*Scaptotrigona mexicana* honey is in high demand in organic food markets and is traditionally used for its medicinal properties, such as treating respiratory problems, digestive disorders, and wounds. This honey is also known for its antimicrobial properties [8]. Scientific studies have confirmed its antimicrobial and antioxidant potential [9]. Honey is fundamentally a unique food product, and its exact chemical composition is related to several different important fields: health, ecology, pollination, food, manufacturing, economy, adulteration, etc. The components in the more traditional *A. mellifera* honey differ significantly from the less studied stingless bee honey. Only recently, in 2021, using ^13^C NMR spectroscopy, the presence of the rare sugar isomer trehalulose was unequivocally demonstrated in the latter [10], and in the present work, we use the same methodology. Up until now, studies on *S. mexicana* honey have mainly focused on physicochemical properties [11]. Chemical composition data are limited to total phenolics, total carotenoids, and identification of lactic acid and 5-(hydroxymethyl)furfural (HMF) [11,12,13]. Our approach is based on ^13^C NMR chemical profiling of *S. mexicana* honey, and provides detailed information on the sugar composition, not only for the usually determined saccharides glucose, fructose, andsucrose, but also for the less abundant, but important for type discrimination, honey sugars such as erlose, panose, maltulose, turanose, trehalulose, and nigerose. Several organic and amino acids as well as some additional honey components can be determined as well.

*S. mexicana* propolis, which is also a valuable bee product, is poorly studied, too. There is only one study of its chemical composition; some flavonoids, chromenes, totarolon, and hydroxycinnamic acids have been identified in it through liquid chromatography with tandem mass spectrometry [14]. Its good antibacterial activity has also been proven [10]. We obtained chemical profiles of *S. mexicana* propolis using the well-established gas chromatography–mass spectrometry approach for propolis profiling. Due to the intricate chemical composition of propolis, GC-MS emerged as the predominant method in the 1980s for the rapid chemical profiling of propolis samples from various geographic locations and plant origins [15]. However, most of the components in propolis are relatively polar, such as flavonoids, phenolic acids, diterpenic acids, etc., which necessitate derivatization (silylation) to improve their volatility and facilitate GC analysis. This circumstance, coupled with the introduction of soft ionization techniques compatible with liquid chromatography in the 1990s, quickly elevated high performance liquid chromatography-diod array detector and high performance liquid chromatography-mass spectrometry as the preferred methods for analyzing propolis alcohol-soluble constituents of propolis [16,17].

Nonetheless, the exceptional resolving capability of capillary GC and the valuable structural insights provided by electron impact mass spectrometry continue to entice researchers to utilize GC-MS, despite the drawbacks associated with derivatization procedures [18,19].

The importance of the bee species on the chemical profile of stingless bee honey and propolis and the specificity of the plant sources used have not yet been clarified in depth [14]. It is generally accepted that the chemical composition of stingless bee honey and propolis depends on the plant sources used and that it is influenced by the available flora near the hives, bee preferences, and climate (altitude and temperature) [20]. Whether species-specific bee preferences or nearby flora and climate are of primary importance in this respect is still debated.

There are contradictory data for honey; for example, Kek et al. [21] claimed that physicochemical and antioxidant parameters can be used for entomological discrimination of stingless bee honeys. On the other hand, Sujanto et al. [22] suggest that “the composition and functional properties of stingless bee honey is differs depending on the source of honey; either influenced by the location of hive or by the species of stingless bee itself.” Using nuclear magnetic resonance (NMR) and chemometrics, Shievano et al. [23] were able to obtain discrimination based on the entomological origin, but only if the limited distribution regions of bee species were taken into consideration.

There are also conflicting studies on propolis. Carneiro et al. [24] demonstrated that *Tetragonisca angustula* bees in different locations collected the same resin to produce propolis, while Velikova et al. [25] found no definite correlation between bee species and propolis origin/chemistry for several bee species.

To determine the relative influence of flora, climate, and bee species-specific preferences, we studied the composition of honey and propolis from two *S. mexicana* meliponaries (or stingless bee yards) in the state of Chiapas, in southern Mexico. The meliponaries were located only 8.5 km from each other, but with an altitude difference of over 150 m. To characterize the chemical variability of stingless bee honey and propolis, samples from 24 colonies were analyzed: 12 from each meliponary. Our results demonstrate that the climate and specific flora around the hives influence the chemical composition of honey and propolis collected by bees of the same species. Furthermore, we addressed the emerging question of whether the saccharide trehalulose can be used as a biomarker for stingless bees’ honey in general [26].

## 2. Materials and Methods

### 2.1. Bee Species and Study Sites

**Figure 1:**
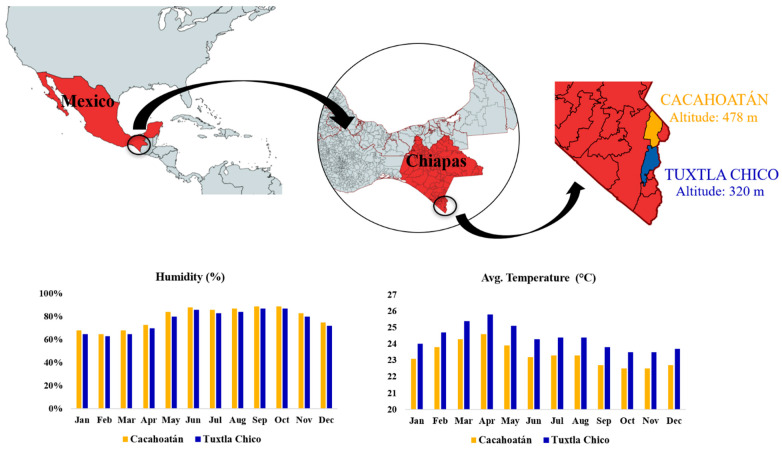
Climatic characteristics and altitude of the two locations.

For the study, 12 × 2 *Scaptotrigona mexicana* honey (Mh) and propolis (Mp) samples were collected from two meliponaries in Mexico in 2021. The bee species was determined by stingless beekeeping technician Miguel Guzmán. Bees were kept in boxes. The meliponaries were located in Tuxtla Chico (T; 14.89° N 92.18° E, 320 msnm) and in Cacahoatán (C; 15.00° N 92.16° E, 478 msnm). Both study sites are located within the Pacific Coastal Plains phytogeographic region. The vegetation consists of a mosaic of secondary strata covered by high evergreen forest. Both study sites are characterized by a warm and humid climate with an annual average temperature of 31 °C, but precipitation varies significantly between sites. Average annual rainfall in Tuxtla Chico is 2488.9 mm, while average annual rainfall in Cacahoatán is 951 mm. In Figure 1, the location and climate of the study sites are visualized.

### 2.2. Propolis Sample Collection

Five grams of propolis was collected from the interior of each of the 24 colonies studied in October/November 2021. Propolis was scraped from the underside of the lid of each colony using sterile gloves and clean serrated knives; fresh extraction equipment was used for each colony to avoid sample contamination. Samples were placed in sterile 50 mL Falcon tubes, labeled, and subsequently stored at 10 °C.

### 2.3. Honey Sample Collection

Twenty milliliters of honey from the interior of each colony was collected using a sterile syringe and stored in an amber glass bottle (120 mL) in May 2021. Fresh extraction equipment was used for each colony to avoid sample contamination. Samples were labeled and stored in a refrigerator at 10 °C.

### 2.4. Honey Sample Preparation

For the preparation of honey samples, 320 mg honey was dissolved in 418 µL of purified water. Then, 187 µL phosphate buffer in D_2_O solution at pH~4.5 were added. The pH was adjusted to 4.2 with small amounts of 0.1M H_3_PO_4_ or 0.1 M NaOH.

### 2.5. NMR Spectroscopy

NMR spectra were acquired on a Bruker NEO 600 spectrometer (Biospin GmbH, Rheinstetten, Germany) at 300.0 ± 0.1 K using a probe head Prodigy. Parameters for ^13^C NMR spectra as used in [27,28] were applied. In addition to sugars, several identified (e.g., meso- and racemic 2,3-butanediol) and 16 other organic components (unknown) were used in the analysis (Table S1).

### 2.6. Propolis Extraction and Sample Preparation

Propolis samples were grated after cooling and extracted with 70% ethanol (1:10, *w/v*) at room temperature, 2 × 24 h. The extracts were filtrated and evaporated to dryness under vacuum. Dry extracts were silylated: about 5 mg of dry extract was mixed with 50 μL dry pyridine and 75 μL N,O-bis(trimethylsilyl)trifluoracetamide (Sigma-Aldrich, Darmstadt, Germany), heated at 80 °C for 20 min, and analyzed by GC-MS.

### 2.7. Total Phenolics

Total phenolic content was determined using the Folin–Ciocalteu method. Each honey sample (2.5 g) was diluted to 25 mL with distilled water. Then, 0.5 mL of the solution was mixed with 2 mL of Folin–Ciocalteu reagent (Merck, Darmstadt, Germany), and 3 mL of 20% sodium carbonate (*w/v*) (Labosi, Paris, France) solution was added. The volume was made up to 25 mL with distilled water. Absorbance at 760 nm was measured after 2 h. Gallic acid (Sigma–Aldrich Chemie, Steinheim, Germany) in a concentration range 28–220 µg/mL was used as standard to obtain the calibration curve. Total phenolic content was expressed in mg of gallic acid equivalents (GAE)/100 g of honey (Table S2).

### 2.8. GC-MS Analysis

GC-MS analysis was performed with a GC coupled to a tandem mass spectrometer GC-MS-TQ 8050 NX Shimadzu (Tokyo, Japan), operating in Q3 scan mode and equipped with a 30 m long, 250 μm i.d., and 0.5 μm film thickness SAPIENS-5MS capillary column (Teknokroma, Barcelona, Spain). The oven temperature was programmed to increase from 75 to 300 °C at a rate of 5 °C/min, with 5 min hold at 75 °C and 25 min hold at 300 °C. Carrier gas He was kept at a constant flow rate of 0.8 mL/min. Pressure was 45.9 kPa, split ratio was 75:1, injector temperature 280 °C, interface temperature 310 °C, and ionization voltage 70 eV. Compounds were identified by computer searches of commercial libraries, comparison with spectra of authentic samples, and data from the literature. The percentage values in Table S3 refer to the percentage of the total ion current (TIC) and are semi-quantitative (semi-quantification was performed by internal normalization.)

### 2.9. Free Radical Scavenging Activity

The radical scavenging activity (RSA) against 2,2-diphenyl-1-picrylhydrazyl (DPPH) was measured as previously described [29]. Each dry extract was dissolved in MeOH to final concentration of 1 mg/mL. Bulgarian propolis was used as a standard (0.1 mg/mL). Then, 2 mL of fresh methanolic DPPH solution (0.1 mM) was mixed with a 100 μL aliquot of each tested sample.

After 30 min storage in a dark place, the absorption decrease was measured at 517 nm (UV-vis spectrophotometer Thermo Scientific Helios gamma). Results were expressed as percentages relative to the control value. The DPPH scavenging activity of the tested samples was calculated using the following equation:

RSA (%) = [(A_0_ − A_S_)/A_0_] × 100 (1)

where A_0_ is the absorbance of the control sample (100 μL MeOH instead of the aliquot volume of the sample) and A_S_ is the absorbance of the tested sample. Each sample was analyzed in triplicate.

### 2.10. Ferric-Reducing Antioxidant Power (FRAP) Assay

The assay was performed as previously published [30], with slight modifications. The FRAP reagent was freshly prepared: 10 parts of 0.3 M acetate buffer (pH 3.6), 1 part of TPTZ (2,4,6-tri(2-pyridyl)-1,3,5-triazine) in 40 mM HCl, and 1 part of 20 mM FeCl_3_·6H_2_O in distilled H_2_O. The FRAP reagent (3 mL) was mixed with the test sample (100 μL solution with a concentration of 1 mg/mL for stingless bees propolis and 0.1 mg/mL for Bulgarian propolis, which was used as a standard), and the mixture was kept 30 min at room temperature in the dark. After that, absorbance was measured at 593 nm against a blank. The FRAP value, in μmol Fe^2+^/L, was calculated from a calibration curve of FeSO_4_·7H_2_O standard solutions. Each sample was analyzed in triplicate (Table S4).

### 2.11. Statistical Analysis

**Figure 2:**
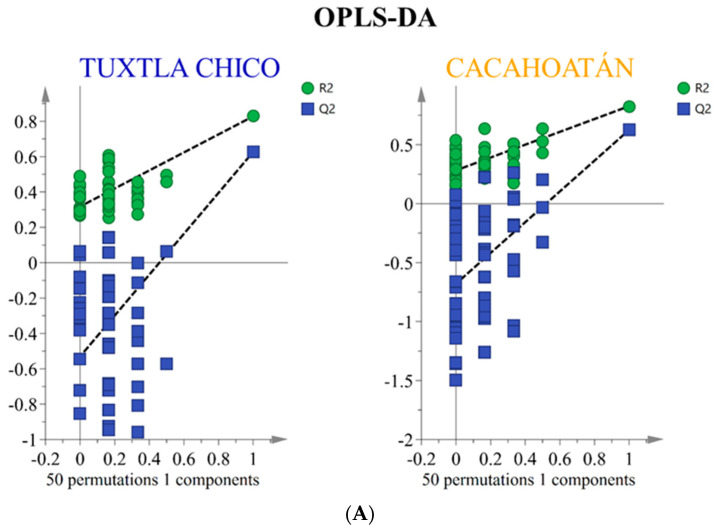

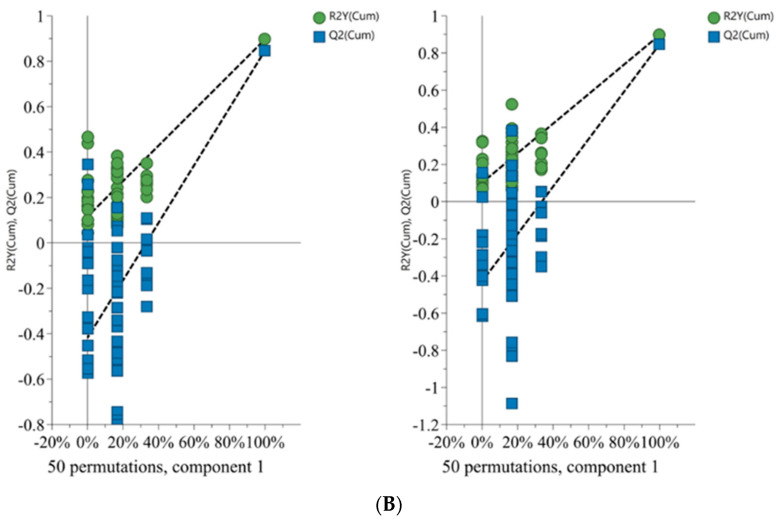
Permutation tests that validate the OPLS-DA models used (**A**) for honey and (**B**) for propolis.

Orthogonal partial least squares discriminant analysis (OPLS-DA) was performed using SIMCA 17.0.2 software (Sartorius Stedim Data Analytics AB, Umetrics, Malmö, Sweden). OPLS-DA is a supervised method, consisting of the separation of a priori given classes of objects, used to obtain the best classification and establish the discriminant models [31,32]. The analysis used semi-quantitative data for compounds observed in 24 samples: 43 compounds for honey and 10 groups of compounds for propolis. In both cases, the samples were divided into two classes (groups) with an equal number of samples in each class. Misclassification tables (Table 1, same for honey and propolis) and permutation tests (Figure 2) were used to validate the established OPLS-DA models and assess their predictive ability. These techniques ensure the reliability and accuracy of the models by evaluating their performance and ability to correctly classify samples from both classes. Nightingale’s diagrams were created using Excel software (Microsoft Office Standard 2019). The chart is typically used to visually represent individual characteristics and allows for a more intuitive understanding of the variation in the two groups.

DPPH and FRAP values were presented as means and standard deviation of triplicates. Pearson’s correlation, one-way ANOVA, and Tukey’s post hoc test at significance levels of *p* < 0.01 and/or *p* < 0.05 were performed with Excel (ChemOffice 2019, PerkinElmer, Shelton, CT, USA).

## 3. Results and Discussion

### 3.1. Chemical Profiles of Honey

For the chemical profiling of the honey samples, we used ^13^C NMR spectroscopy as previously described [27,28].

**Figure 3:**
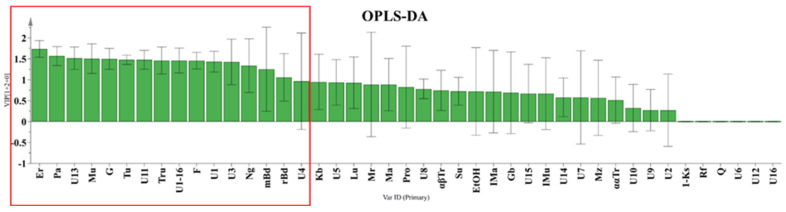
VIP values used in the OPLS-DA analysis for honey samples substances: Er—erlose, Pa—panose, Mu—maltulose, G—glucose, Tu—turanose, Tru—trehalulose, F—fructose, Ng—nigerose, m/r Bd—meso and racemic 2,3-butanediol. Other quantified compounds: Kb—kojibiose, Lu—leucrose; Mr—maltotriose, Ma—maltose, Pro—proline, αβTr—αβ-trehalose, Su—sucrose, Ima—isomaltose, Gb—gentiobiose, IMu—isomaltulose, Mz—melezitose, ααTr—αα-trehalose, 1-Ks—1-kestose, Rf—raffinose, Q—quercitol, U—unknown).

**Figure 4:**
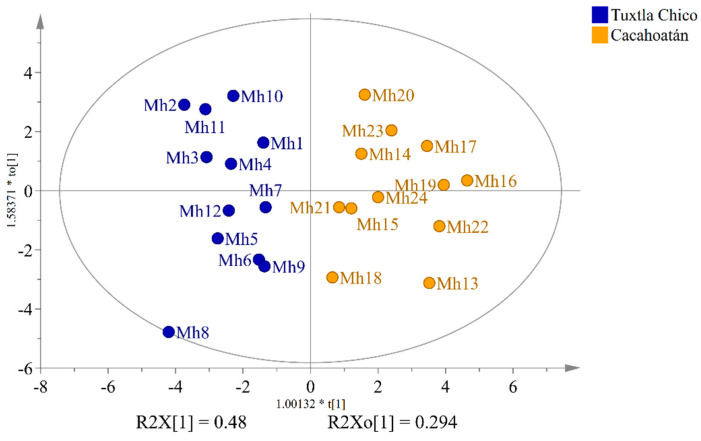
OPLS-DA score plot of honey samples from two regions, Tuxtla Chico and Cacahoatán, Mexico.

To investigate the differences in the honey sugar profiles (Table S1) originating from two different geographical regions, the widely used supervised statistical method orthogonal partial least squares discriminant analysis (OPLS-DA) was used to analyze the obtained quantitative data from ^13^C NMR spectra. By using two-component OPLS-DA analysis, VIP (variable importance in projection) values were obtained (Figure 3). They revealed 16 statistically significant compounds (shown in the red box) to differentiate between *S. mexicana* honey from Tuxtla Chico and Cacahoatán. Subsequent use of an OPLS-DA based on these 16 compounds (R2X(cum) = 0.862; R2Y(cum) = 0.827; Q2(cum) = 0.626) visually demonstrated significant differences in the composition of the honey samples, presented in Figure 4.

**Figure 5:**
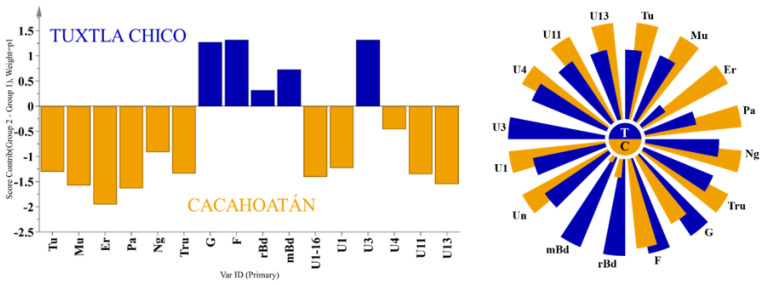
OPLS-DA contribution plot (left) and Nightingale’s diagram (right) for the average content of significant substances (Tu—turanose, Mu—maltulose, Er—erlose, Pa—panose, Ng—nigerose, Tru—trehalulose, G—glucose, F—fructose, m/r Bd—meso and racemic 2,3-butanediol, Un—unknown) in honey samples from two regions, Tuxtla Chico (T) and Cacahoatán (C).

Honey samples from Tuxtla Chico contained higher concentrations of glucose (17.4–25.2 g/100 g) and fructose (26.8–30.6 g/100 g) than those from Cacahoatán, where glucose concentrations varied from 14.6 to 20.0 g/100 g and fructose from 24.3 to 28.1 g/100 g. Cacahoatán honey samples were characterized by a rich composition of di- and trisaccharides, including maltulose, turanose, trehalulose, erlose, and panose. It should be noted that the concentration of erlose in all samples from this region exceeded 1 g/100 g, while only one sample from Tuxtla Chico, Mh7, demonstrated such a high level with a concentration of 1.2 g/100 g. The differences between the two honey groups are illustrated using the contribution plot and the Nightingale’s diagram (Figure 5). These variations can be attributed to the distinct nectar sources used by bees in each geographical region. The differences in altitudes and average temperatures between the two regions (Figure 1) contribute to differences in plant species and their nectar composition.

An interesting observation is the significant presence of trehalulose in all honey samples—over 6.7 g/100 g. Recently, this unusual reducing sugar, an α-(1→1) glucose-fructose isomer of sucrose, was found in the honey of different stingless bee species from Asia, Australia, Africa, and South America, and it has been suggested that trehalulose may be a marker for its authenticity [10,33]. The presence of trehalulose is considered beneficial due to its known low glycemic index, acariogenic properties, and antioxidant activity [33].

In a series of feeding experiments with Australian stingless bees, Hungerford et al. [34] demonstrated that the conversion of fed sucrose resulted in trehalulose (64–72%) with less erlose (18–23%) and fructose (9–12%). On the other hand, feeding solutions of glucose/fructose mixtures (1:1) did not result in the formation of trehalulose/erlose. This disaccharide is thought to be the result of certain enzymatic processes due to microorganisms in the stingless bee nest and not to come from the floral sources of the nectar. Thus, Hungerford et al. [34] speculated that stingless bees with access to high-sucrose floral nectar would produce honey high in trehalulose. This hypothesis is supported by our results, as the honey from Tuxtla Chico, which has higher fructose and glucose content, has lower amounts of trehalulose.

However, there are still insufficient published data to conclude that trehalulose is a universal marker for stingless bee honey. Recent work from Malaysia [35] and some unpublished data for honey from Malaysia and Indonesia [36] indicate that this hypothesis needs verification in future studies.

The total phenolic content of the honey samples was also studied. Values varied between 61.5 and 111 mg GAE/100 g (Table S2). This is consistent with previous studies of different stingless bee honeys [37]. Our data are also similar to data for *A. mellifera* (honey bee) honey, which displays a wide range of values (18.9 to 177 mg GAE/100 g), depending on the geographical region of origin [38,39]. It has been demonstrated that the Folin–Ciocalteu method can be considered suitable for the purpose of antioxidant potency evaluation [40]. This suggests that the *S. mexicana* honey has similar antioxidant potential to *A. mellifera* honey.

The results for the samples from the two locations did not demonstrate statistically significant differences: honey samples from Tuxtla Chico contained an average of 79.9 ± 12 mg GAE/100 g, while honey samples from Cacahoatán contained an average of 74.8 ± 10 mg GAE/100 g (*p* = 0.279).

### 3.2. Chemical Profiles of Propolis

The chemical composition of the 70% ethanol extracts of the propolis samples was analyzed using gas chromatography-mass spectrometry (GC-MS) after silylation. More than 40 individual compounds belonging to various chemical classes were identified such as phenolic lipids (cardols, cardanols, and anacardic acids), diterpenes, triterpenes, and lignans. The complete chemical profiles of all samples can be found in Table S3.

**Figure 6:**
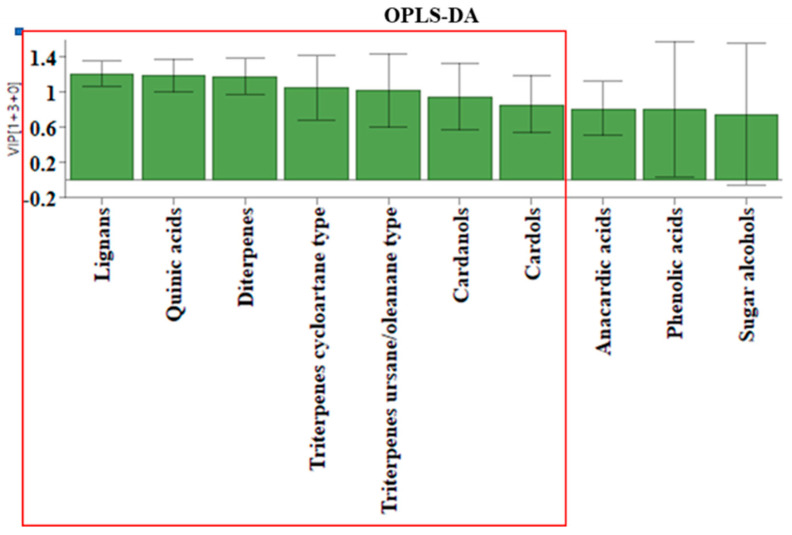
VIP values used in the OPLS-DA analysis for the propolis samples are presented in the red box.

**Figure 7:**
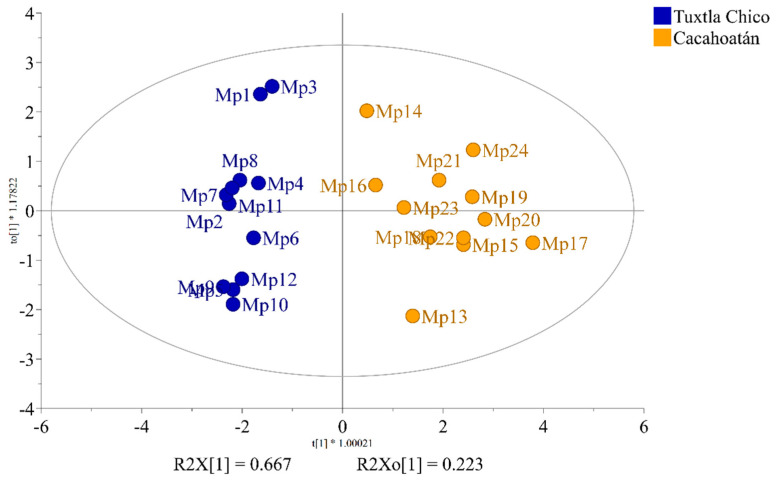
OPLS-DA score plot of propolis samples from two regions, Tuxtla Chico and Cacahoatán, Mexico.

**Figure 8:**
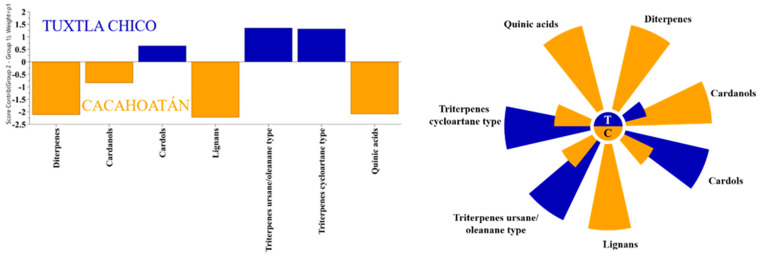
OPLS-DA contribution plot (**left**) and Nightingale’s diagram (**right**) for the average content of significant substances in propolis samples from two regions, Tuxtla Chico (T) and Cacahoatán (C).

To determine whether the propolis samples could be distinguished according to their geographical origin, a two-component OPLS-DA analysis was applied analogously to the honey samples. The relative content of the compounds as groups, according to structural type rather than the percentage content as individual substances, was used for the analysis. Of the ten different groups of compounds that were identified, only seven proved to be statistically significant for distinguishing between *S. mexicana* propolis from Tuxtla Chico and Cacahoatán regions according to the VIP values presented in Figure 6. This model, based on seven groups of compounds (R2X(cum) = 0.984; R2Y (cum) = 0.897; Q2(cum) = 0.847), demonstrates the formation of two distinct groups of samples visualized in Figure 7. The variables responsible for the distinction between the two groups and their contribution are presented in Figure 8.

All samples from both locations contained the typical *Mangifera indica* chemical markers: cycloartane type triterpenes (cycloartenol, mangiferolic, and isomangiferolic acids) and the group of phenolic lipids, mainly cardols (alk(en)yl resorcinols) [41]. However, the two sample groups also demonstrated a significant difference. While 11 of the 12 samples from the Tuxtla Chico region contained almost no secondary metabolites other than mango tree compounds, those from the Cacahoatán region contained kaurane type diterpenes(mainly kaurenoic acid), dibenzylbutanediol lignans (dihydrocubebin and 3,4-methylenedioxy secoisolariciresinol), and small amounts of quinic acid isomers. The difference in the chemical profiles of the two groups of samples is shown in Figure 8. Our results differ from published data [20]: flavonoids, chromenes, and totarolon were not identified in our samples.

These results are a solid indication that the bees in the Cacahoatán meliponary collected resin from another botanical source in addition to the mango latex, because the propolis produced by them contained diterpenes, lignans, and quinic acids that were not found in the Tuxtla Chico samples. The only exception is sample Mp-11 (Tuxtla Chico), in which diterpenes and lignans were detected in significantly lower amounts. The assumption that all three groups of constituents (diterpenes, lignans, and quinic acids) that differed from the Tuxtla Chico samples came from the same source plant is supported by the statistically significant correlation between the concentration of lignans and diterpenes (R = 0.818, *p* < 0.01) and between the lignans and quinic acids concentration (R = 0.859, *p* < 0.01). The predominance of a single additional resin source in these samples is surprising, however, given that bees were observed entering Cacahoatán colonies with resins of various different colors (red, brown, orange, white) twice a week for a year. To the best of our knowledge, no plant resin, exudate, or latex has been reported to contain a combination of all these substances. Indeed, in many cases, especially in Gymnosperm resins, lignans and diterpenes occur simultaneously, but a material with a combination of kaurenoic acid and kaurene type diterpenes with dibenzylbutanediol lignans is not yet known. The only information on such a co-occurrence refers to *Aristolochia* species [42,43]. However, these plants do not produce any substance on their surface to attract stingless bees and serve as a source of resinous material for the bees. The botanical origin of these compounds has not yet been revealed; additional observations of bee behavior can be very helpful in determining their source. This plant must be attractive to resin foragers; as already mentioned, one of the samples from Tuxtla Chico contains small amounts of the same diterpenes and lignans, although it is probably located far from their source.

The antioxidant activity of the extracts was tested using the DPPH radical scavenging activity test and a FRAP test. The results are presented in Table S4. DPPH radical inhibition values for both groups were relatively low and did not show statistically significant differences, in contrast to FRAP. No correlation was observed between DPPH and FRAP values of all samples, but this was expected [41]. The samples from Cacahoatán had a higher ferric-reducing antioxidant potential than Tuxtla Chico ones, and the difference was statistically significant at *p* < 0.05 (*p* = 0.0425). The presence of lignans probably accounts for this difference. This assumption is supported by the strong positive correlation between FRAP and lignan concentration in this group of samples (R = 0.8991, *p* < 0.01). It is interesting to note that dihydrocubebin has been found to possess significant antituberculosis activity [44].

## 4. Conclusions

In conclusion, our results indicate the importance of geographical location, in particular through plant origin and climate, on the composition of honey and propolis produced by the same stingless bee species. The observed substantial differences in the chemical composition of propolis and honey of *S. mexicana* from two relatively close locations definitively support the conclusion that the bee species cannot be considered the most important factor in determining the chemistry of these products. Propolis composition indicates that the bees visit different resin sources at the two locations. The significant difference we observed between the honeys from the two regions is not consistent with the recent conclusion [45] that entomological origin is the main factor that determines the characteristics of honey, and that the floral origin is only the secondary factor. Further studies are needed to clarify whether species-specific bee preferences or local flora and climate are more important for the chemical composition of stingless bee honey and propolis.

## Figures and Tables

**Table 1:** Misclassification table for the honey/propolis from the two regions.

	Members	Correct	Tuxtla Chico	Cacahoatán	No Class (YPred ≤ 0)
**Tuxtla Chico**	12	100%	12	0	0
**Cacahoatán**	12	100%	0	12	0
**No class**	0		0	0	0
Total	24	100%	12	12	0
Fisher’s prob.	3.7 × 10^−7^				

## Data Availability

The data of the current study are available from the corresponding authors on reasonable request.

## Supplementary Materials

The following supporting information can be downloaded at: https://www.mdpi.com/article/10.3390/foods12173317/s1, Table S1: Chemical composition of the honey samples studied (in g/100 g); Table S2: Total phenolic content of *Scaptotrigona mexicana* honey; Table S3: Chemical composition of the investigated propolis samples (GC-MS after silylation; %TIC); Table S4: DPPH and FRAP for *Scaptotrogona mexicana* propolis.

## References

[1] Roubik D.W., Smith B.H., Carlson R.G. (1987). Formic acid in caustic cephalic secretions of stingless bee, *Oxytrigona* (Hymenoptera: Apidae). J. Chem. Ecol..

[2] Hrncir M., Jarau S., Barth F.G.J. (2016). Stingless bees (Meliponini): Senses and behavior. J. Comp. Physiol. A.

[3] Roubik D.W. (1989). Ecology and Natural History of Tropical Bees.

[4] Sanches M.A., Pereira A.M.S., Serrão J.E. (2017). Pharmacological actions of extracts of propolis of stingless bees (Meliponini). J. Apicult. Res..

[5] Reyes-González A., Camou-Guerrero A., Del-Val E., Ramírez M.I., Porter-Bolland L. (2020). Biocultural diversity loss: The decline of native stingless bees (Apidae: Meliponini) and local ecological knowledge in Michoacán, Western México. Hum. Ecol..

[6] Arnold N., Zepeda R., Vásquez V.D., Maya E.M.A. (2018). Las Abejas sin Aguijón y su Cultivo en Oaxaca, México.

[7] Gutierrez A., Obregon F.H., Jones W.R. (2002). Optimum brood size for artificial propagation of the stingless bee, *Scaptotrigona mexicana*. J. Apic. Res..

[8] Vit P., Medina M., Eunice Enríquez M. (2004). Quality standards for medicinal uses of Meliponinae honey in Guatemala, Mexico and Venezuela. Bee World.

[9] Jimenez M., Beristain C.I., Azuara E., Mendoza M.R., Pascual L.A. (2016). Physicochemical and antioxidant properties of honey from *Scaptotrigona mexicana* bee. J. Apic. Res..

[10] Popova M., Gerginova D., Trusheva B., Simova S., Tamfu A.N., Ceylan O., Clark K., Bankova V. (2021). A preliminary study of chemical profiles of honey, cerumen, and propolis of the African stingless bee *Meliponula ferruginea*. Foods.

[11] Garay L.A.L., Téllez L.I.T., Merino F.C.G., Oliva A.C., Sato J.A.P., Ruíz J.S. (2023). Physicochemical properties of *Scaptotrigona mexicana* honey from the Highlands of Veracruz, Mexico. Ecosistemas y Recursos Agropecuarios.

[12] Grajales-Conesa J., Vandame R., Santiesteban-Hernández A., López-García A., Guzmán-Díaz M. (2018). Propiedades fisicoquímicas y antibacterianas de mieles de abejas sin aguijón del Sur de Chiapas, México. IBCiencias.

[13] Vit P., Rojas L.B., Usubillaga A., Aparicio R., Meccia G., Muiño M.A.F., Sancho M.T. (2011). Presence of lactic acid and other semivolatil compound in Meliponini honeys. RINHRR.

[14] Grajales-Conesa J., Elías-Chirino J., Lozano-Guzmán E., Moreno-Cruz F., Albores-Flores V., López-García A. (2021). Stingless bees propolis antimicrobial activity in combination with garlic, *Allium sativum* (Amaryllidaceae). Rev. Biol. Trop..

[15] Greenaway W., Scaysbrook T., Whatley F.R. (1990). The composition and plant origin of propolis: A report of work at Oxford. Bee World.

[16] Ahn M.R., Kumazawa S., Usui Y., Nakamura J., Matsuka M., Zhu F., Nakayama T. (2007). Antioxidant activity and constituents of propolis collected in various areas of China. Food Chem..

[17] Mohamed W.A.S., Ismail N.Z., Muhamad M., Omar E.A., Samad N.A., Ooi J.P., Mohamad S. (2022). Q-TOF LC-MS compounds evaluation of propolis extract derived from Malaysian stingless bees, *Tetrigona apicalis*, and their bioactivities in breast cancer cell, MCF7. Saudi J. Biol. Sci..

[18] Popova M., Silici S., Kaftanoglu O., Bankova V. (2005). Antimicrobial activity of Turkish propolis and its qualitative and quantitative chemical composition. Phytomedicine.

[19] Garzoli S., Maggio F., Vinciguerra V., Rossi C., Donadu M.G., Serio A. (2023). Chemical Characterization and Antimicrobial Properties of the Hydroalcoholic Solution of *Echinacea purpurea* (L.) Moench. and Propolis from Northern Italy. Molecules.

[20] Souza B., Roubik D., Barth O., Heard T., Enríquez E., Carvalho C., Villas-Bôas M.L., Locatelli J., Persano-Oddo L., Almeida-Muradian L. (2006). Composition of stingless bee honey: Setting quality standards. Interciencia.

[21] Kek S.P., Chin N.L., Tan S.W., Yusof Y.A., Chua L.S. (2017). Molecular identification of honey entomological origin based on bee mitochondrial 16S rRNA and COI gene sequences. Food Control.

[22] Sujanto I.S.R., Ramly N.S., Abd Ghani A., Huat J.T.Y., Alias N., Ngah N. (2021). The composition and functional properties of stingless bee honey: A review. Malaysian, J. Anal. Sci..

[23] Schievano E., Mammi S., Menegazzo I., Vit P., Pedro S.R.M., Roubik D. (2013). Nuclear Magnetic Resonance as a Method to Predict the Geographical and Entomological Origin of Pot-Honey. Pot-Honey: A Legacy of Stingless Bees.

[24] Carneiro M.J., López B.G., Lancellotti M., Franchi G.C., Nowill A.E., Sawaya A.C.H.F. (2016). Evaluation of the chemical composition and biological activity of extracts of *Tetragonisca angustula* propolis and *Schinus terebinthifolius* Raddi (Anacardiaceae). J. Apic. Res..

[25] Velikova M., Bankova V., Tsvetkova I., Kujumgiev A., Marcucci M.C. (2000). Antibacterial *ent*-kaurene from Brazilian propolis of native stingless bees. Fitoterapia.

[26] Zawawi N., Zhang J., Hungerford N.L., Yates H.S., Webber D.C., Farrell M., Tinggi U., Bhandari B., Fletcher M.T. (2022). Unique physicochemical properties and rare reducing sugar trehalulose mandate new international regulation for stingless bee honey. Food Chem..

[27] Gerginova D., Dimova D., Simova S. (2017). Preliminary NMR and chemometric study of pine jams used as medicinal remedies. Bulg. Chem. Commun..

[28] Gerginova D., Simova S., Popova M., Stefova M., Stanoeva J.P., Bankova V. (2020). NMR Profiling of North Macedonian and Bulgarian Honeys for Detection of Botanical and Geographical Origin. Molecules.

[29] Tomasina F., Carabio C., Celano L., Thomson L. (2012). Analysis of two methods to evaluate antioxidants. Biochem. Mol. Biol. Educ..

[30] Benzie I., Devaki M., Apak R., Capanoglu E., Shahidi F. (2018). The ferric reducing/antioxidant power (FRAP) assay for non-enzymatic antioxidant capacity: Concepts, procedures, limitations and applications. Measurement of Antioxidant Activity and Capacity: Recent Trends and Applications.

[31] Chaodi K., Yingying Z., Mingyue Z., Jing Q., Wentao Z., Jin G., Wenping G., Yingying L. (2022). Screening of specific quantitative peptides of beef by LC–MS/MS coupled with OPLS-DA. Food Chem..

[32] Arendse E., Fawole O.A., Magwaza L.S., Nieuwoudt H., Opara U.L. (2018). Evaluation of biochemical markers associated with the development of husk scald and the use of diffuse reflectance NIR spectroscopy to predict husk scald in pomegranate fruit. Sci. Hortic..

[33] Fletcher M.T., Hungerford N.L., Webber D., Carpinelli de Jesus M., Zhang J., Stone I.S., Blanchfield J.T., Zawawi N. (2020). Stingless bee honey, a novel source of trehalulose: A biologically active disaccharide with health benefits. Sci. Rep..

[34] Hungerford N.L., Zhang J., Smith T.J., Yates H.S., Chowdhury S.A., Carter J.F., Carpinelli de Jesus M., Fletcher M.T. (2021). Feeding sugars to stingless bees: Identifying the origin of trehalulose-rich honey composition. J. Agric. Food Chem..

[35] Ng W.-J., Sit N.-W., Ooi P.A.-C., Ee K.-Y., Lim T.-M. (2021). Botanical Origin Differentiation of Malaysian Stingless Bee Honey Produced by *Heterotrigona itama* and *Geniotrigona thoracica* Using Chemometrics. Molecules.

[36] Simova S. (2023). Trehalulose in Stingless Bees.

[37] Ali H., Abu Bakar M.F., Majid M., Muhammad N., Lim S.Y. (2020). In vitro anti-diabetic activity of stingless bee honey from different botanical origins. Food Res..

[38] Wilczyńska A. (2014). Effect of filtration on colour, antioxidant activity and total phenolics of honey. LWT—Food Sci. Technol..

[39] Pauliuc D., Dranca F., Oroian M. (2020). Antioxidant activity, total phenolic content, individual phenolics and physicochemical parameters suitability for Romanian honey authentication. Foods.

[40] Rumpf J., Burger R., Schulze M. (2023). Statistical evaluation of DPPH, ABTS, FRAP, and Folin-Ciocalteu assays to assess the antioxidant capacity of lignins. Int. J. Biol. Macromol..

[41] Ediriweera M.K., Tennekoon K.H., Samarakoon S.R. (2017). A review on ethnopharmacological applications, pharmacological activities, and bioactive compounds of *Mangifera indica* (Mango). Evid.-Based Complement. Alternat. Med..

[42] Zhang G., Shimokawa S., Mochizuki M., Kumamoto T., Nakanishi W., Watanabe T., Ishikawa T., Matsumoto K., Tashima K., Horie S. (2008). Chemical constituents of *Aristolochia constricta*: Antispasmodic effects of its constituents in guinea-pig ileum and isolation of a diterpeno–lignan hybrid. J. Nat Prod..

[43] Pereira A.O., Avila J.M., do Carmo G., Siqueira F.S., Campos M.M., Back D.F., Morel A.F., Dalcol I.I. (2018). Chemical composition, antimicrobial and antimycobacterial activities of *Aristolochia triangularis* Cham. from Brazil. Ind. Crop.Prod..

[44] Koirala N., Modi B., Subba R.K., Panthi M., Xiao J., Dua K., Nammi S., Chang D., Chellappan D.K., Gupta G., Collet T. (2021). Medicinal Plants in Targeting Tuberculosis II. Medicinal Plants for Lung Diseases.

[45] May-Canché I., Moguel-Ordoñez Y., Valle-Mora J., González-Cadenas R., Toledo-Núñez B., Arroyo-Rodríguez L., Piana L., Rémy Vandame R. (2022). Sensory and physicochemical analysis of honeys of nine stingless bee species of Mexico and Guatemala. J. Food Sci. Technol..

