# Supplementary material for: The Chemical Composition of Scaptotrigona mexicana Honey and Propolis Collected in Two Locations: Similarities and Differences
Source: Foods. 2023 Sep 3;12(17):3317. doi: 10.3390/foods12173317 (PMC10486931; doi:10.3390/foods12173317)
Supplement: Supplementary file 1 [file foods-12-03317-s001.zip › foods-2556599-supplementary.pdf]

Table S1. Chemical composition of the honey samples studied (in g/100 g)

	Tuxtla Chico														
	**Mh1**	**Mh2**	**Mh3**	**Mh4**	**Mh5**	**Mh6**	**Mh7**	**Mh8**	**Mh9**	**Mh10**	**Mh11**	**Mh12**	**min**	**max**	**avg.**
**G**	23.64	25.24	23.50	23.61	20.22	18.85	18.07	18.29	17.40	21.83	24.83	18.52	17.40	25.24	21.17
**F**	27.60	30.57	28.99	29.27	28.93	27.74	27.27	27.74	26.81	29.29	29.73	28.16	26.81	30.57	28.51
**Gb**	0.00	0.00	0.00	0.00	0.00	0.00	0.12	0.00	0.00	0.00	0.20	0.14	0.00	0.20	0.04
**Su**	0.18	0.00	0.11	0.10	0.00	0.10	0.15	0.16	0.14	0.11	0.10	0.11	0.00	0.18	0.10
**IMa**	0.69	0.57	0.73	0.74	0.68	0.55	0.68	0.66	0.57	0.63	0.84	0.49	0.49	0.84	0.65
**IMu**	0.83	0.59	0.00	0.60	0.55	0.46	0.82	0.56	0.60	0.39	1.05	0.67	0.00	1.05	0.59
**Kb**	1.98	1.75	2.05	1.83	2.48	2.56	2.61	2.74	2.57	2.40	1.52	2.88	1.52	2.88	2.28
**Lu**	0.27	0.20	0.24	0.23	0.26	0.20	0.25	0.23	0.26	0.26	0.31	0.17	0.17	0.31	0.24
**Ma**	1.17	1.34	1.64	1.23	2.00	2.03	1.97	2.51	2.12	1.94	1.11	2.37	1.11	2.51	1.79
**Mu**	3.68	2.63	3.21	3.44	3.74	4.16	3.60	4.10	4.12	2.70	3.06	3.49	2.63	4.16	3.49
**Ng**	0.83	1.01	0.93	0.83	1.22	1.16	1.05	0.94	1.07	1.32	0.72	1.16	0.72	1.32	1.02
**ααTr**	0.00	0.00	0.08	0.00	0.00	0.07	0.00	0.00	0.00	0.05	0.00	0.00	0.00	0.08	0.02
**αβTr**	0.42	0.33	0.39	0.40	0.36	0.46	0.40	0.44	0.42	0.31	0.34	0.34	0.31	0.46	0.38
**Tru**	8.94	6.71	7.86	8.39	9.27	9.96	9.77	10.29	10.61	6.98	7.50	8.72	6.71	10.61	8.75
**Tu**	2.43	2.15	2.57	2.38	3.18	3.61	3.24	3.81	3.68	2.49	1.95	3.29	1.95	3.81	2.90
**Er**	0.32	0.19	0.27	0.24	0.23	0.95	1.19	0.52	0.80	0.73	0.15	0.84	0.15	1.19	0.54
**1-Ks**	0.00	0.00	0.00	0.00	0.00	0.00	0.00	0.00	0.00	0.00	0.00	0.00	0.00	0.00	0.00
**Mr**	0.31	0.27	0.39	0.25	0.45	0.56	0.34	0.32	0.43	0.36	0.00	0.45	0.00	0.56	0.34
**Mz**	0.15	0.00	0.07	0.00	0.00	0.00	0.00	0.00	0.00	0.00	0.00	0.00	0.00	0.15	0.02
**Pa**	0.24	0.23	0.33	0.33	0.39	0.52	0.39	0.39	0.50	0.29	0.14	0.42	0.14	0.52	0.35
**mBd**	0.03	0.05	0.03	0.06	0.02	0.00	0.02	0.02	0.00	0.00	0.06	0.00	0.00	0.06	0.02
**rBd**	0.04	0.02	0.02	0.01	0.01	0.00	0.01	0.00	0.00	0.00	0.03	0.00	0.00	0.04	0.01
**Pro**	0.03	0.00	0.00	0.03	0.03	0.01	0.02	0.02	0.04	0.03	0.04	0.03	0.00	0.04	0.02
**U1-16**	3.82	2.92	3.58	3.74	4.00	4.12	4.00	4.23	4.24	3.00	3.26	3.64	2.92	4.24	3.71

	Cacahoatán														
	**Mh13**	**Mh14**	**Mh15**	**Mh16**	**Mh17**	**Mh18**	**Mh19**	**Mh20**	**Mh21**	**Mh22**	**Mh23**	**Mh24**	**min**	**max**	**avg.**
**G**	14.58	19.16	18.56	16.40	17.85	17.47	16.84	20.04	17.13	16.31	18.67	16.78	14.58	20.04	17.48
**F**	25.67	28.04	27.66	26.45	27.12	26.71	26.97	28.13	26.67	24.26	27.47	26.64	24.26	28.13	26.82
**Gb**	0.10	0.17	0.00	0.04	0.00	0.11	0.12	0.16	0.06	0.07	0.00	0.00	0.00	0.17	0.07
**Su**	0.18	0.00	0.08	0.09	0.22	0.14	0.08	0.10	0.13	0.21	0.10	0.16	0.00	0.22	0.12
**IMa**	0.62	0.70	0.52	0.56	0.66	0.80	0.67	0.56	0.66	0.67	0.68	0.73	0.52	0.80	0.65
**IMu**	0.72	0.80	0.93	0.73	0.45	0.55	0.61	0.41	0.74	0.64	0.57	0.67	0.41	0.93	0.65
**Kb**	2.48	2.34	2.34	2.49	2.38	2.01	2.48	2.34	2.48	2.53	2.32	2.37	2.01	2.53	2.38
**Lu**	0.30	0.20	0.28	0.24	0.26	0.31	0.27	0.18	0.25	0.30	0.32	0.24	0.18	0.32	0.26
**Ma**	2.02	1.92	1.76	1.90	1.85	1.43	1.84	2.00	1.94	2.07	1.79	2.02	1.43	2.07	1.88
**Mu**	5.24	4.12	4.59	4.97	4.34	4.93	4.92	3.50	4.04	4.80	4.16	4.35	3.50	5.24	4.50
**Ng**	1.04	1.46	1.31	1.25	1.31	1.05	1.35	1.68	1.12	1.31	1.09	1.24	1.04	1.68	1.27
**ααTr**	0.00	0.02	0.05	0.11	0.00	0.00	0.00	0.00	0.00	0.00	0.00	0.06	0.00	0.11	0.02
**αβTr**	0.39	0.37	0.36	0.43	0.40	0.41	0.36	0.33	0.38	0.39	0.34	0.40	0.33	0.43	0.38
**Tru**	12.65	9.42	10.26	11.82	10.46	11.38	11.05	8.49	10.63	11.86	9.89	11.04	8.49	12.65	10.74
**Tu**	4.71	3.42	3.67	4.00	3.82	3.42	4.23	3.14	3.70	4.25	3.36	3.70	3.14	4.71	3.79
**Er**	1.72	1.00	1.12	1.52	1.40	1.11	1.44	1.06	1.47	1.30	1.06	1.28	1.00	1.72	1.29
**1-Ks**	0.00	0.00	0.00	0.00	0.00	0.00	0.00	0.00	0.00	0.00	0.00	0.00	0.00	0.00	0.00
**Mr**	0.53	0.28	0.33	0.29	0.37	0.41	0.39	0.44	0.57	0.36	0.39	0.41	0.28	0.57	0.40
**Mz**	0.09	0.05	0.06	0.00	0.07	0.09	0.00	0.07	0.06	0.00	0.00	0.04	0.00	0.09	0.04
**Pa**	0.73	0.44	0.42	0.66	0.56	0.65	0.58	0.42	0.53	0.67	0.57	0.52	0.42	0.73	0.56
**mBd**	0.00	0.00	0.00	0.00	0.00	0.00	0.00	0.01	0.00	0.03	0.01	0.01	0.00	0.03	0.01
**rBd**	0.00	0.00	0.00	0.00	0.00	0.00	0.00	0.01	0.00	0.01	0.02	0.01	0.00	0.02	0.00
**Pro**	0.03	0.02	0.03	0.03	0.04	0.03	0.02	0.02	0.03	0.03	0.05	0.03	0.02	0.05	0.03
**U1-16**	5.27	4.04	4.35	4.80	4.38	4.78	4.75	3.58	4.31	4.90	4.21	4.44	3.58	5.27	4.48

Acronyms: F—Fructose; G—Glucose; Gb—Gentiobiose; Su—Sucrose; Ima—Isomaltose; IMu—Isomaltulose; Kb—Kojibiose; Lu—Leucrose; Ma—Maltose; Mu—Maltulose; Ng—Nigerose; Tru—Trehalulose; Tu—Turanose; ααTr—αα-Trehalose; αβTr—αβ-Trehalose; Er—Erlose; 1-Ks—1-Kestose; Mr—Maltotriose; Mz—Melezitose; Pa—Panose; mBd—meso-2,3butanediol; rBd—racemic 2,3butanediol; Pro—Proline; U—Unknown.

Table S2. Total phenolic content of *Scaptotrigona mexicana* honey.

**Honey sample**	**TPC (mg GAE/100 g)**
Mh1	90.9±2.8
Mh2	73.4±0.2
Mh3	80.9±1.5
Mh4	86.4±0.4
Mh5	84.2±0.7
Mh6	73.0±0.8
Mh7	70.5±2.1
Mh8	76.4±1.3
Mh9	72.6±0.4
Mh10	67.0±3.5
Mh11	111.0±1.4
Mh12	72.4±1.2
Mh13	64.6±1.8
Mh14	84.4±1.8
Mh15	67.5±2.1
Mh16	61.5±1.4
Mh17	63.2±2.3
Mh18	64.6±0.4
Mh19	77.2±1.9
Mh20	82.2±3.2
Mh21	88.4±1.8
Mh22	89.8±1.6
Mh23	72.9±1.9
Mh24	81.3±3.3

Table S3. Chemical composition of the investigated propolis samples (GC-MS after silylation; %TIC).

	Tuxtla Chico											
**Compound**	Mp1	Mp2	Mp3	Mp4	Mp5	Mp6	Mp7	Mp8	Mp9	Mp10	Mp11	Mp12
Sugar alcohols	**2.9**	**6.8**	**2.0**	**2.1**	**4.8**	**1.3**	**1.9**	**0.5**	**9.5**	**8.3**	**1.3**	**0.4**
Glycerol	1.9	2.1	1.4	1.5	3.0	0.8	1.2	0.3	6.3	4.3	0.5	0.3
Arabitol	1.0	4.3	0.6	0.5	1.2	0.5	0.7	0.2	2.6	2.7	0.8	0.1
Pinitol	-	Tr.	Tr.	Tr.	0.4	Tr.	Tr.	-	0.3	0.6	Tr.	-
Mannitol	-	0.4	-	0.1	0.2	Tr.	Tr.	-	0.3	0.7	Tr.	Tr.
Phenolic acids	**0.3**	**0.3**	**Tr.**	**0.3**	**0.2**	**Tr.**	**Tr.**	**0.2**	**1.1**	**1.5**	**Tr.**	**0.1**
Hydroxybenzoic acid	Tr.	0.1	Tr.	0.1	Tr.	Tr.	Tr.	0.2	0.8	0.2	Tr.	Tr.
Hydroxyphenyl acetic acid	0.3	Tr.	Tr.	0.2	0.2	Tr.	Tr.	Tr.	0.3	1.3	Tr.	0.1
p-Coumaric acid	Tr.	0.2	-	Tr.	-	-	Tr.	-	-	-	-	-
Caffeic acid	-	Tr.	-	-	-	-	-	-	-	-	-	-
Diterpenes	**-**	**-**	**-**	**-**	**-**	**-**	**-**	**-**	**-**	**-**	**1.2**	**-**
Kaurenal	-	-	-	-	-	-	-	-	-	-	-	-
Kaurenol	-	-	-	-	-	-	-	-	-	-	-	-
Kaurenoic acid	-	-	-	-	-	-	-	-	-	-	1.0	-
Hydroxucaurenoic acid	-	-	-	-	-	-	-	-	-	-	Tr.	-
Acetoxycaurenoic acid	-	-	-	-	-	-	-	-	-	-	0.2	-
Cardanols	**1.3**	**0.6**	**1.4**	**0.3**	**Tr.**	**Tr.**	**0.3**	**0.2**	**Tr.**	**Tr.**	**0.4**	**Tr.**
Cardanol C17:2	0.4	0.4	0.4	0.2	Tr.	Tr.	0.2	0.2	Tr.	Tr.	0.2	Tr..
Cardanol C17:1	0.1	0.2	0.2	0.1	Tr.	Tr.	0.1	Tr.	Tr.	Tr.	0.2	Tr.
Cardanol C19:1	0.8	Tr.	0.8	Tr.	-	Tr.	Tr.		Tr.	Tr.	Tr.	Tr.
Cardols	**19.3**	**12.6**	**18.4**	**9.5**	**3.6**	**5.9**	**13.3**	**13.2**	**6.3**	**2.7**	**14.2**	**2.9**
Cardol C15:1	0.6	0.7	0.5	0.2	Tr.	0.2	0.4	0.3	0.3	0.1	0.9	0.2
Cardol C15:0	1.4	1.2	1.5	0.7	0.3	0.5	1.1	1.0	0.6	0.2	0.1	Tr.

Cardol C17:2	7.0	5.0	8.4	3.6	1.1	1.9	5.4	5.1	2.0	0.8	5.0	0.8
Cardol C17:1	1.3	0.7	1.1	0.6	0.2	0.5	1.1	0.9	0.5	0.2	0.7	0.2
Cardol C17:1 (isomer)	1.3	0.8	1.4	0.6	0.3	0.4	0.9	0.9	0.4	0.2	0.9	0.3
Cardol C17:1 (isomer)	4.5	2.7	4.8	2.5	0.9	1.3	3.9	3.3	1.7	0.8	3.7	0.8
Cardol C17:0	0.2	Tr.	Tr.	Tr.	0.2	0.3	0.2	Tr.	0.1	Tr.	0.3	Tr.
Cardol C19:1	3.0	1.5	0.4	1.3	0.6	0.8	0.3	1.7	0.7	0.4	2.6	0.6
Lignans	**-**	**-**	**-**	**-**	**-**	**-**	**-**	**-**	**-**	**-**	**0.8**	**-**
Dihydrocubebin	-	-	-	-	-	-	-	-	-	-	0.8	-
3.4-Methylenedioxy secoisolariciresinol	-	-	-	-	-	-	-	-	-	-	-	-
Anacardic acids	**2.6**	**4.0**	**8.4**	**3.1**	**1.2**	**1.7**	**5.5**	**5.1**	**1.9**	**1.0**	**5.7**	**1.4**
Anacardic acid C15:0	0.2	0.1	0.5	0.2	Tr.	0.1	0.4	0.4	0.2	Tr.	0.4	Tr.
Anacardic acid C15:2	1.2	1.8	3.3	1.2	0.4	0.6	2.1	2.0	0.7	0.4	2.0	0.4
Anacardic acid C15:1	1.2	1.2	1.9	1.0	0.4	0.6	1.8	1.5	0.7	0.4	1.7	0.4
Anacardic acid C17:1		0.9	2.7	0.7	0.4	0.4	1.2	1.2	0.3	0.2	1.6	0.6
Triterpenes ursane/oleanane type	**10.6**	**8.1**	**11.9**	**19.0**	**13.1**	**20.8**	**12.0**	**17.9**	**12.4**	**12.7**	**10.8**	**14.7**
β-Amyrenone	0.8	0.1	0.3	1.4	0.7	2.1	1.0	1.0	1.4	1.4	0.1	1.6
β-Amyrin	4.1	3.0	3.6	4.4	2.9	3.7	3.2	4.7	3.6	2.3	3.3	2.7
α-Amyrenone	1.2	0.9	1.2	3.0	1.4	5.6	2.4	2.4	1.0	3.0	1.0	3.5
α-Amyrin	4.5	3.8	6.8	7.9	4.8	4.6	3.4	8.3	4.4	3.0	5.3	3.3
Amyrin acetate	-	0.3	-	2.3	3.3	4.8	2.0	1.5	2.0	3.0	1.1	3.6
Triterpenes cycloartane type	**16.4**	**13.2**	**16.5**	**16.1**	**10.1**	**12.6**	**15.3**	**15.6**	**9.0**	**8.7**	**14.9**	**11.5**
Cycloartenol	7.5	5.7	5.4	8.2	6.1	7.6	5.4	6.2	5.7	4.9	5.4	6.1
Mangiferolic acid	1.7	1.6	2.2	2.0	1.3	1.6	2.5	2.4	0.7	1.3	2.5	2.2
Isomaniferolic acid	7.2	5.9	8.9	5.9	2.7	3.4	7.4	7.0	2.6	2.5	7.0	3.2
Others												
Ethylamine	1.0	1.1	3.2	1.0	0.9	0.2	1.5	1.1	1.2	2.7	1.1	0.8

	Cacahoatán											
	**Mp13**	**Mp14**	**Mp15**	**Mp16**	**Mp17**	**Mp18**	**Mp19**	**Mp20**	**Mp21**	**Mp22**	**Mp23**	**Mp24**
Sugar alcohols	**1.1**	**0.7**	**0.6**	**1.0**	**0.8**	**0.6**	**2.1**	**1.7**	**1.5**	**3.1**	**5.6**	**1.2**
Glycerol	0.7	0.5	0.3	0.7	0.5	0.4	1.4	1.0	0.8	1.7	1.5	1.0
Arabitol	0.4	0.2	0.3	0.3	0.3	0.2	0.6	0.7	0.7	1.3	1.1	0.2
Pinitol	-	-	-	-	-	-	0.1	-	-	0.1	Tr.	-
Phenolic acids	**0**	**0**	**0**	**0**	**0**	**0**	**0.2**	**0**	**0**	**0**	**0.2**	**0**
p-coumaric acid	-	-	-	-	-	-	0.2	-	-	-	-	-
Diterpenes	**47.1**	**14.5**	**39**	**21.8**	**44.7**	**32.4**	**31.9**	**37.5**	**27.1**	**34.1**	**23.3**	**29.2**
Kaurenal	2.2	0.6	1.4	0.7	1.1	1.0	1.0	1.2	0.8	1.2	0.7	1.0
Kaurenol	4.3	1.2	2.9	1.2	2.6	2.1	2.0	2.8	1.7	2.6	1.4	1.9
Kaurenoic acid	32.6	9.1	26.3	14.9	27.4	22.1	21.8	25.6	18.5	23.3	16.4	20.0
Hydroxucaurenoic acid	1.9	0.9	1.9	1.2	2.5	1.8	1.8	1.7	1.5	1.7	1.2	1.7
Acetoxycaurenoic acid	6.1	2.7	6.5	3.8	11.1	5.4	5.2	6.2	4.6	5.3	3.6	4.6
Cardanols	**0.3**	**3.6**	**1.8**	**2.1**	**1.6**	**2.1**	**1.8**	**1.4**	**2.2**	**-**	**-**	**-**
Cardanol C17:2	Tr.	0.8	0.6	0.4	05	0.7	0.6	0.6	0.4	0.3	0.3	0.6
Cardanol C17:1	Tr.	0.7	0.2	0.5	0.6	-	0.6	0.4	0.6	0.6	0.5	0.7
Cardanol C19:1	-	-	-	-	-	-	-	0.2	0.2	-	-	0.2
Cardanol C19:1 (isomer)	0.3	2.1	1.0	1.2	0.5	1.4	0.6	0.5	1.0	0.6	0.8	1.1
Cardols	**1.4**	**11.5**	**3.6**	**9.9**	**1.5**	**5.4**	**3.6**	**2.6**	**6**	**2.7**	**5.1**	**6**
Cardol C15:1	-	0.5	Tr.	0.3	-	0.2	Tr.	0.2	0.5	0.1	0.2	0.2
Cardol C15:0	-	1.0	0.3	0.7	-	0.4	0.2	0.2	-	0.2	0.4	0.5
Cardol C17:2	0.6	4.4	1.7	4.5	0.8	2.2	1.7	1.0	3.0	1.1	2.2	2.3
Cardol C17:1	0.2	1.1	0.3	0.7	0.3	0.4	0.4	0.3	0.5	-	0.4	0.5
Cardol C17:1 (isomer)	0.2	1.0	0.3	0.6	0.2	0.4	0.4	0.2	1.4	0.3	0.4	0.6
Cardol C17:1 (isomer)	0.4	3.3	1.0	3.0	0.5	1.8	0.9	0.7	0.6	0.2	1.5	1.9
Cardol C17:0	Tr.	0.2	Tr.	0.1	Tr.	Tr.	Tr.	Tr.	Tr.	0.8	Tr.	Tr.

Anacardic acids	**Tr.**	**5.1**	**1.5**	**2.4**	**0.4**	**2.3**	**1.2**	**0.8**	**2.2**	**1.6**	**2.0**	**1.4**
Anacardic acid C15:2	Tr.	1.9	0.9	1.4	0.2	0.9	0.4	0.4	1.2	0.6	0.8	0.5
Anacardic acid C15:1	Tr.	1.6	0.6	Tr.	0.1	0.8	0.6	0.3	0.8	0.6	0.7	0.5
Anacardic acid C17:1	-	1.3	-	1.0	0.1	0.6	0.2	0.1	0.2	0.4	0.5	0.4
Anacardic acid C15:0	-	0.3	-	-	-	-	-	-	-	-	-	-
Lignans	**16.2**	**7.8**	**19.8**	**11.6**	**15.5**	**17.1**	**18.5**	**21.2**	**16.4**	**19.3**	**13.2**	**17.3**
Dihydrocubebin	15.4	7.1	18.5	10.9	23.2	16.0	17.4	19.7	15.3	18.0	12.4	16.3
3,4-Methylenedioxy secoisolariciresinol	0.8	0.7	1.3	0.7	2.3	1.1	1.1	1.5	1.1	1.3	0.8	1.0
Triterpenes ursane/oleanane type	**5.1**	**8.7**	**3.3**	**9.4**	**2.3**	**7.7**	**7.9**	**5.2**	**8.4**	**6.2**	**10.9**	**7.8**
β-Amyrenone	0.5	0.6	0.4	0.9	0.2	0.7	0.8	0.5	0.2	0.7	0.7	0.8
β-Amyrin	0.8	3.2	1.3	3.3	0.6	2.0	2.2	1.3	2.4	2.2	3.0	2.8
α-Amyrenone	1.1	1.0	0.6	1.2	0.4	1.3	0.9	0.6	1.0	0.8	1.0	0.9
α-Amyrin	2.1	3.5	1.0	4.0	0.6	2.4	2.0	1.2	2.6	1.6	3.6	3.0
Amyrin acetate	0.6	0.4	-	-	0.5	1.3	2.0	1.6	2.2	0.9	2.6	0.3
Triterpenes cycloartane type	**3.6**	**15.1**	**5.0**	**9.3**	**2.1**	**7.1**	**6.8**	**4.6**	**8.5**	**4.8**	**8.9**	**9.6**
Cycloartenol	2.6	5.1	2.3	5.8	0.9	3.4	3.7	2.8	4.1	3.2	4.8	5.1
Mangiferolic acid	0.4	3.8	1.1	0.8	0.5	1.4	2.0	0.6	1.6	1.6	1.4	1.6
Isomaniferolic acid	0.6	6.2	1.6	2.7	0.7	2.3	1.1	1.0	2.8	-	2.7	2.9
Others												
Ethylamine	1.0	0.1	1.2	1.0	1.1	1.2	1.3	1.2	1.1	1.2	1.2	1.1
Quinic acid	Tr.	0.3	0.8	0.3	1.1	0.5	0.7	0.6	0.5	0.7	0.5	0.7
Quinic acid (isomer)	0.8	0.4	1.1	0.6	1.5	1.0	1.0	0.9	0.8	1.1	0.7	1.0

Tr. – traces (< 0.1);

Table S4. DPPH and FRAP for propolis of *Scaptotrigona mexicana*[a]

Sample	DPPH (%RSA)	FRAP (µmol Fe^{2+}/L)
Mp1	7.16 ± 0.01	178 ± 4
Mp2	5.37 ± 0.19	165 ± 9
Mp3	5.34 ± 0.27	148 ± 1
Mp4	4.35 ± 0.46	173 ±1
Mp5	1.76 ± 0.18	189 ± 9
Mp6	2.05 ± 0.19	171 ± 8
Mp7	4.66 ± 0.11	172 ± 11
Mp8	5.65 ± 0.17	212 ± 10
Mp9	8.50 ± 0.29	281 ± 6
Mp10	3.98 ± 0.15	186 ±4
Mp11	15.34 ± 0.28	149 ± 3
Mp12	13.27 ± 0.07	109 ± 5
Mp13	17.69 ± 0.07	345 ± 9
Mp14	17.14 ± 0.10	248 ± 4
Mp15	17.47 ± 0.17	334 ± 11
Mp16	8.16 ± 0.09	275 ± 14
Mp17	8.80 ± 0.11	337 ± 3
Mp18	6.97 ± 0.09	265 ± 9
Mp19	6.36 ± 0.26	247 ± 4
Mp20	7.06 ± 0.11	300 ± 9
Mp21	7.69 ± 0.33	292 ± 41
Mp22	7.72 ± 0.19	289 ± 3
Mp23	6.87 ± 0.24	279 ± 2
Mp24	7.02 ± 0.33	303 ±5
BG[b]	17.01 ± 0.21	411 ± 1

[a] Tested concentration 1.0 mg/mL.

[b] Bulgarian *Apis mellifera* propolis. tested concentration 0.1 mg/mL.
